# Supplementary material for: Personalized genome assembly for accurate cancer somatic mutation discovery using tumor-normal paired reference samples
Source: Genome Biol. 2022 Nov 9;23:237. doi: 10.1186/s13059-022-02803-x (PMC9648002; doi:10.1186/s13059-022-02803-x)
Supplement: Supplementary file 1 — Additional file 1. Included all the supplementary tables for this manuscript. [file 13059_2022_2803_MOESM1_ESM.pdf]

Personalized genome assembly for accurate cancer somatic mutation discovery using cancer-normal paired reference samples

Chunlin Xiao[1*], Zhong Chen[2], Wanqiu Chen[2], Cory Padilla[3], Michael Colgan[4], Wenjun Wu[5], Li-Tai Fang[6], Tiantian Liu[2], Yibin Yang[5], Valerie Schneider[1], Charles Wang[2*], and Wenming Xiao[4*]

Supplementary Tables

Table S1: Whole genome sequencing data of HCC1395 and HCC1395BL by multiple sequencing platforms used in this study.

Dataset	Sequencing center	Usage	HCC1395		HCC1395BL	
			# Reads	Total base pairs (Depths)	# Reads	Total base pairs (Depths)
Illumina HiSeq [ref 5-6]	Fudan (FD)	Polishing, unitig assembly, SNVs/SVs variant calling	3,403,236,080	510,485,412,000 (170.16x)	3,511,999,736	526,799,960,400 (175.59x)
10X Genomics Chromium [ref 5-6]	Fudan (FD)	Assembly; Scaffolding	3,206,110,814	480,916,622,100 (160.30x)	3,222,458,614	483,368,792,100 (161.12x)
PacBio Sequel [ref 5-6]	CSHL	Primary Contig assembly, phasing, variant calling	17,265,341	140,649,790,223 (46.88x)	17,671,082	160,626,774,754 (53.54x)
Hi-C	Dovetail	Scaffolding	NA	NA	1,432,923,164	214,938,474,600 (71.64x)
Oxford Nanopore	Loma Linda University	Phasing	NA	NA	11,585,856	47,006,385,877 (15.66x)

Table S2: Summary of the *de novo* assemblies for the two cell lines using PacBio long reads and 10X Genomics linked reads.

	**HCC1395BL (10X_supernova)**	**HCC1395 (10X_supernova)**	**HCC1395BL (PacBio_canu)**	**HCC1395 (PacBio_canu)**
# contigs (>= 0 bp)	21,450	23,195	2,900	5,351
Total length (>= 0 bp)	2,893,054,450	2,871,887,885	2,905,219,353	2,853,495,799
# contigs (>= 10,000 bps)	3,416	4,666	2,828	4,949
Total length (>= 10,000 bps)	2,837,767,582	2,812,618,703	2,904,842,414	2,851,315,749
Largest contig	107,564,373	47,962,721	62,208,403	25,523,694
GC (%)	40.89	40.96	40.91	40.95
N50	30,593,498	11,455,133	13,480,407	3,338,913
L50	27	70	57	210
# contigs (>= 10,000 bps) matched GRCh38	3,176	4,449	2,526	4,627
# bps of contig (>= 10,000 bps) matched GRCh38	2,833,045,730	2,808,106,025	2,888,438,712	2,836,167,093
# cumulative bps matches on GRCh38	2,787,709,216	2,754,283,911	2,867,274,440	2,820,906,016
# novel contigs (>= 10,000 bps)	240	217	302	322
# bps of novel contigs (>= 10,000 bps)	4,721,852	4,512,678	16,403,702	15,148,656
RefSeq NMs mapped (95+% alignment covered)	48,061 (96.02%)	47,128 (94.15%)	49,287 (98.41%)	47,619 (95.13%)
RefSeq NRs mapped (95+% alignment covered)	14,423 (92.78%)	14,165 (91.12%)	15,115 (97.24%)	14,717 (94.67%)

Table S3: Summary of genome phasing using WhatsHap with PacBio reads alone and reads from combinations of PacBio and Oxford Nanopore (ONT).

	PacBio reads	PacBio + ONT reads
# Blocks	6,368	3,204
Sum block length (bps)	2,421,024,137	2,543,961,355
Longest block (bps)	6,378,479	20,455,447
# Phased	3,134,631	3,135,816
# Hets in VCF	3,172,233	3,172,233

Table S4: Summary of RefSeq genes and transcripts mapping onto HCC1395BL_v1.0 assembly using BLAT. Genes from ChrY and all pseudogenes were excluded from this analysis. For comparison, the same set of RefSeq sequences were mapped onto GRCh38.

	# RefSeq NM	# genes for RefSeq NM	# RefSeq NR	# genes for RefSeq NR
# Input (excluding chrY, and all pseudo genes)	49,844	19,325	14,132	10,061
# Found on HCC1395BL_v1.0 (blat minIdentity=92)	49,838	19,322	14,131	10,060
# Not Found on HCC1395BL_v1.0	6	3	1	1
# Found 50%+ coverage & 95%+ identity on HCC1395BL_v1.0	49,798 (99.91%)	19,303 (99.89%)	14,120 (99.92%)	10,049 (99.88%)
# Found 95%+ coverage & 95%+ identity on HCC1395BL_v1.0	49,482 (99.27%)	19,164 (99.17%)	13,978 (98.91%)	9,958 (98.98%)
# Found 50%+ coverage & 95%+ identity on GRCh38	49,825 (99.96%)	19,318 (99.96%)	14,128 (99.97%)	10,058 (99.97%)
# Found 95%+ coverage & 95%+ identity on GRCh38	49,669 (99.65%)	19,232 (99.52%)	14,044 (99.37%)	10,002 (99.41%)

Table S5: Summary of somatic SNVs/Indels (FDN123 for normal cell line, and FDT123 for tumor cell line) detected by Strelka2 and MuTect2 on GRCh38 and HCC1395BL assemblies.

	**HCC1395BL_v1.0 as reference**			**GRCh38 as reference**		
	Strelka2	MuTect2	Strelka2 / MuTect2 intersection	Strelka2	MuTect2	Strelka2 / MuTect2 intersection
# SNVs	57,314	52,137	43,285	46,742	49,676	41,669
# Indels (1~20 bps)	3,463	5,224	2,096	2,455	4,373	1,729
# Total	60,777	57,361	45,381	49,197	54,049	43,398

Table S6: Summary of mapping 41,669 GRCh38-based SNVs (intersection of Strelka2/MuTect2 SNVs) to *de novo* assembly HCC1395BL_v1.0, and their functional annotation using ANNOVAR.

	Mapped on de novo assembly WITH overlapping Strelka2/MuTect2 SNVs		Mapped on de novo assembly WITHOUT overlapping Strelka2/MuTect2 SNVs	Not-mapped on *de novo* assembly	Total
	No mismatch in flanking	Mismatch in flanking			
UTR3	363	28	2	1	394
UTR5	107	2	1	1	111
downstream	323	36	7	2	368
exonic	442	28	5	2	477
intergenic	17698	2112	480	135	20425
intronic	12726	1297	115	56	14194
ncRNA_exonic	264	24	3	2	293
ncRNA_intronic	4479	432	62	19	4992
upstream	360	34	7	1	402
splicing	11	2	0	0	13
**Total # SNVs**	**36,773 (88.25%)**	**3,995 (9.59%)**	**682 (1.64%)**	**219 (0.52%)**	**41,669 (100%)**

Table S7: KEGG pathway enrichment analysis of 71 genes overlapped with the 1,017 novel SNVs detected with HCC1395BL_v1.0 as a reference. Shown here are the top 10 enriched pathways with the enriched gene counts versus the total genes in each pathway, P-value, “Odds Ratio” (zScore), and the genes involved.

Term	Overlap	P-value	Odds Ratio	Genes
Axon guidance	4/181	0.00393343	6.662281811	EPHA6;ABLIM2;EFNA5;SSH2
Basal transcription factors	2/45	0.01114736	13.40478598	GTF2H2C;GTF2H2
Nucleotide excision repair	2/47	0.01211671	12.80772947	GTF2H2C;GTF2H2
Aldosterone synthesis and secretion	2/98	0.04747244	5.988224638	CYP21A2;ATP1B3
Other types of O-glycan biosynthesis	1/22	0.07529437	13.54285714	POMT2
Mannose type O-glycan biosynthesis	1/23	0.07858068	12.92662338	POMT2
Proximal tubule bicarbonate reclamation	1/23	0.07858068	12.92662338	ATP1B3
Apoptosis	2/143	0.09171747	4.067838421	PTPN13;LMNB1
Glyoxylate and dicarboxylate metabolism	1/30	0.10126467	9.802955665	ACSS1
Propanoate metabolism	1/32	0.10764406	9.169585253	ACSS1

Table S8: Summary of PCR validations using Sanger sequencing.

Primer	Primer sequence	Size	Status
scaffold_3_49125164_F1	TTCTGTTGCAGTCGGTCACT	309	Confirmed
scaffold_3_49125164_R1	GGCAGGAGAACTGCTTGAAC		
scaffold_4_51676090_F1	CCAAACTCAAGCATAGCCAAT	317	Confirmed
scaffold_4_51676090_R1	GCCTATGTAACAAAGTCTTCAGGA		
scaffold_4_78466556_F1	ATCTTGACCCATCCTTGCAT	305	Confirmed
scaffold_4_78466556_R1	GGACCTGACAGTTTTACTGCTG		
scaffold_11_7213726_F1	TGGTGGAAAGTGGGAGTACA	245	Confirmed
scaffold_11_7213726_R1	GAGAAGCCAGAGGTTGTTGG		
scaffold_14_24934345_F1	TGGACACAGGAAGGGGAATA	354	Confirmed
scaffold_14_24934345_R1	GCCTACTCAGGCCTCCTCTT		
scaffold_17_32882935_F1	CAAGCCAAATTTGACCACAA	301	Confirmed
scaffold_17_32882935_R1	AGCATTAGGGTGGCTATCAGG		
scaffold_17_32958026_F1	GGGATCGCTATCTCTCACCA	364	Confirmed
scaffold_17_32958026_R1	TGCATTTGTGAATTGTGCTG		
scaffold_35_28067196_F1	TGACGCTGCTCTGTCTTTTG	234	Confirmed
scaffold_35_28067196_R1	CAAGGCTGTCTGTTCACCAA		
scaffold_20_687304_F1	ACATGCCTGTAATCCCACCT	333	Partially Confirmed
scaffold_20_687304_R1	TGAAGCCACTTCTTGTGGTG		
scaffold_19_2641776_F1	ATGAATCTGGGTGCTCCTGT	250	Unconfirmed
scaffold_19_2641776_R1	AGGAAACCCATCTCATGTGC		

Table S9: Somatic SVs detected by 4 somatic SV callers (GRIDSS2, Manta, Delly, and novoBreak) with HCC1395BL_v1.0 reference were all increased, as compared to GRCh38, with short-read sequencing data from a paired sample (FDT123/FDN123).

	Including TRA		Excluding TRA		SVs called by 2 or more callers	
	# SVs increased	% SVs increased	# SVs increased	% SVs increased	# SVs increased	% SVs increased
**GRIDSS2**	82	8.82%	32	4.85%	34	4.17%
**Manta**	189	17.83%	72	9.89%	50	5.78%
**Delly**	54	10.71%	36	7.94%	34	7.21%
**novoBreak**	86	11.46%	26	5.26%	11	2.09%

Table S10: Somatic SVs detected in two or more replicates by GRIDSS2, Manta, Delly, and novoBreak with HCC1395BL_v1.0 reference were all increased, as opposed to GRCh38, with short-read sequencing data from 12 replicates.

	Including TRA		Excluding TRA	
	# SVs increased	% SVs increased	# SVs increased	% SVs increased
**GRIDSS2**	105	11.93%	38	5.87%
**Manta**	147	14.32%	48	6.72%
**Delly**	42	6.46%	25	4.25%
**novoBreak**	85	9.49%	25	4.28%

Table S11: Manual inspections in IGV demonstrated that 9 of 11 gene-overlapping somatic deletions, which were supported by two or more short-read SV callers and were no GRCh38-based SVs being mapped in their locations on HCC1395BL_v1.0, were confirmed with in-read deletions from PacBio long-reads in tumor cell line, but not in normal cell line.

Scaffold Name	SV_Start	SV_End	SV Type	SV Length (bps)	Gene Symbols	Repeat_Types in deleted sequences by repeatmasker	# Somatic SV callers supporting SV using short-reads	PacBio long-read supports (IGV)
scaffold_14	28524259	28540166	DEL	15,907	PALM2-AKAP2	LINE/L1, SINE/Alu, SINE/MIR, DNA/hAT-Charlie, DNA/TcMar-Tigger	4	Uncertain
scaffold_16	34011167	34012303	DEL	1136	IQCH	LINE/L1, SINE/Alu	2	Confirmed with in-read deletions
scaffold_17	32976348	32976659	DEL	311	CCDC91	SINE/Alu	3	Confirmed with in-read deletions
scaffold_20	2320477	2320665	DEL	188	SRPK2	/	2	Confirmed with in-read deletions
scaffold_26	608684	608920	DEL	236	ZMYM2	/	2	Confirmed with in-read deletions
scaffold_31	16874991	16875249	DEL	258	UBTF	Simple_repeat, Low_complexity	4	Confirmed with in-read deletions
scaffold_44	5949987	5950071	DEL	84	DDIAS	DNA/TcMar-Tigger	2	Confirmed with in-read deletions
scaffold_49	9367688	9641734	DEL	274,046	OR4M2, OR4N4	LINEs, SINEs, LTR etc	4	Uncertain
scaffold_6	44613899	44613956	DEL	57	MED12L	Simple_repeat	3	Confirmed with in-read deletions
scaffold_73	790019	790931	DEL	912	NBPF14, PDE4DIP	LINE/L2, LTR/ERVL-MaLR	3	Confirmed with in-read deletions
scaffold_83	194853	195151	DEL	298	PDXDC1	SINE/Alu	2	Confirmed with in-read deletions

Table S12: Summary of somatic SV counts in tumor sample (HCC1395) with PacBio long-reads using two aligners (PBMM2 and NGMLR) and two SV callers (PBSV and Sniffles2), and with contigs using paftools and Assemblytics with HCC1395BL_v1.0 reference as compared to GRCh38.

	**GRCh38**	**HCC1395BL_v1.0**
PBMM2_PBSV	1,187	1,365
PBMM2_Sniffles2	1,424	1,563
NGMLR_PBSV	1,098	1,268
NGMLR_Sniffles2	1,339	1,572
Paftools	1,169	1,334
Assemblytics	9,16	1,234

Table S13: Summary of mapping 744 GRCh38-based SVs (supported by 3 or more calling methods) to HCC1395BL_v1.0 reference and their functional annotation using ANNOVAR.

	# SVs Mapped with matching HCC1395BL_v1.0-based SVs	# SVs Mapped but without HCC1395BL_v1.0-based SVs	# SVs Un-mapped on HCC1395BL_v1.0
UTR3	3	1	0
UTR5	2	0	1
downstream	4	1	0
exonic	51	2	1
intergenic	225	63	43
intronic	170	52	23
ncRNA_exonic	24	1	2
ncRNA_intronic	48	8	11
upstream	4	1	3
Total	**531**	**129**	**84**

Table S14: The top 10 enriched pathways from KEGG pathway enrichment analysis for 86 genes that overlapped with 91 HCC1395BL_v1.0-based SVs without GRCh38-based SVs in their corresponding locations.

**Term**	**Overlap**	**P-value**	**Odds Ratio**	**Genes**
Glutamatergic synapse	4/114	0.001484099	8.782261641	GRM7;GNG7;DLGAP1;ADCY5
MicroRNAs in cancer	5/310	0.010865986	3.968629832	RPTOR;ABCC1;ST14;TNC;EZR
PI3K-Akt signaling pathway	5/354	0.01831768	3.460504439	RPTOR;FLT4;GNG7;TNC;EIF4B
Amyotrophic lateral sclerosis	5/364	0.020388235	3.362392104	PRKN;DNAH12;DNAH10;DNAH17;OPTN
Mitophagy	2/68	0.034664591	7.16017316	PRKN;OPTN
Renin secretion	2/69	0.035601512	7.052949538	ACE;ADCY5
Thyroid hormone synthesis	2/75	0.041424795	6.471298108	TPO;ADCY5
Gastric acid secretion	2/76	0.042428057	6.383526384	EZR;ADCY5
RNA transport	3/186	0.046214618	3.897096583	EIF2B3;EIF4B;EIF3B
Pathways of neurodegeneration	5/475	0.053962236	2.553716837	PRKN;DNAH12;DNAH10;DNAH17;OPTN
