# Supplementary material for: Personalized genome assembly for accurate cancer somatic mutation discovery using tumor-normal paired reference samples
Source: Genome Biol. 2022 Nov 9;23:237. doi: 10.1186/s13059-022-02803-x (PMC9648002; doi:10.1186/s13059-022-02803-x)
Supplement: Supplementary file 2 — Additional file 2. Included all the supplementary figures for this manuscript. [file 13059_2022_2803_MOESM2_ESM.pdf]

Personalized genome assembly for accurate cancer somatic mutation discovery using cancer-normal paired reference samples

Chunlin Xiao[1*], Zhong Chen[2], Wanqiu Chen[2], Cory Padilla[3], Michael Colgan[4], Wenjun Wu[5], Li-Tai Fang[6], Tiantian Liu[2], Yibin Yang[5], Valerie Schneider[1], Charles Wang[2*], and Wenming Xiao[4*]

Supplementary Figures

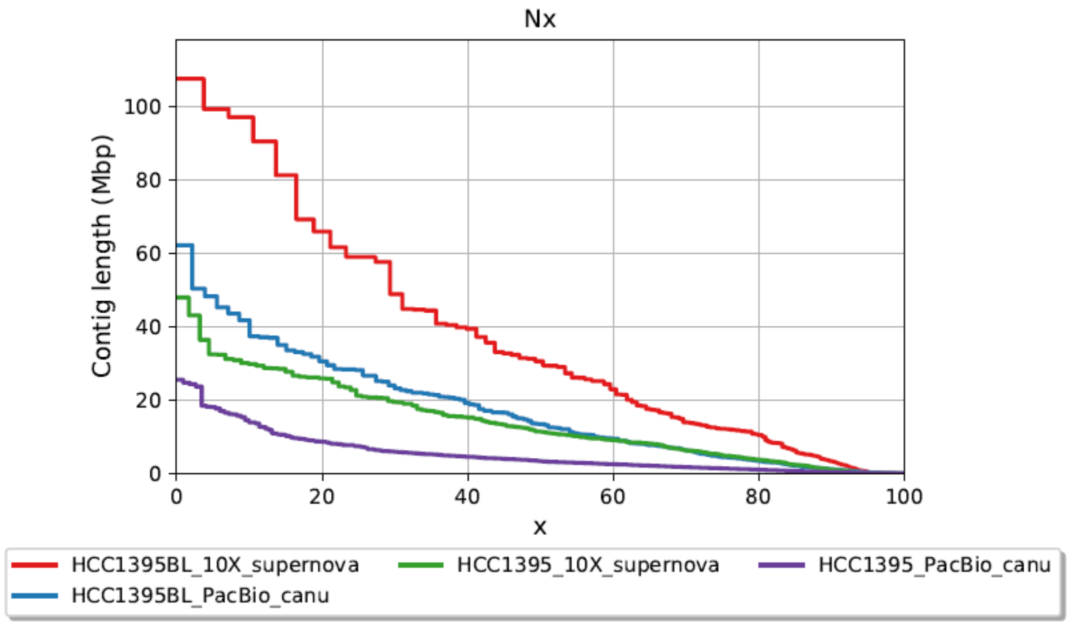

Fig. S1. Nx plot of the assemblies from PacBio long reads using canu and 10X Genomics linked reads using supernova.

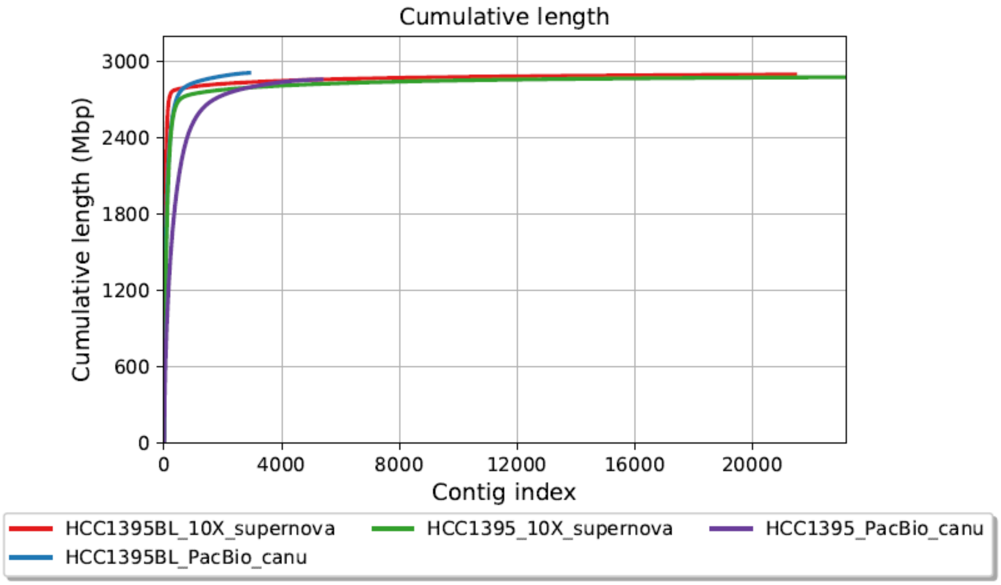

Fig. S2. Cumulative length plot of the assemblies from PacBio long reads using canu and 10X Genomics linked reads using supernova.

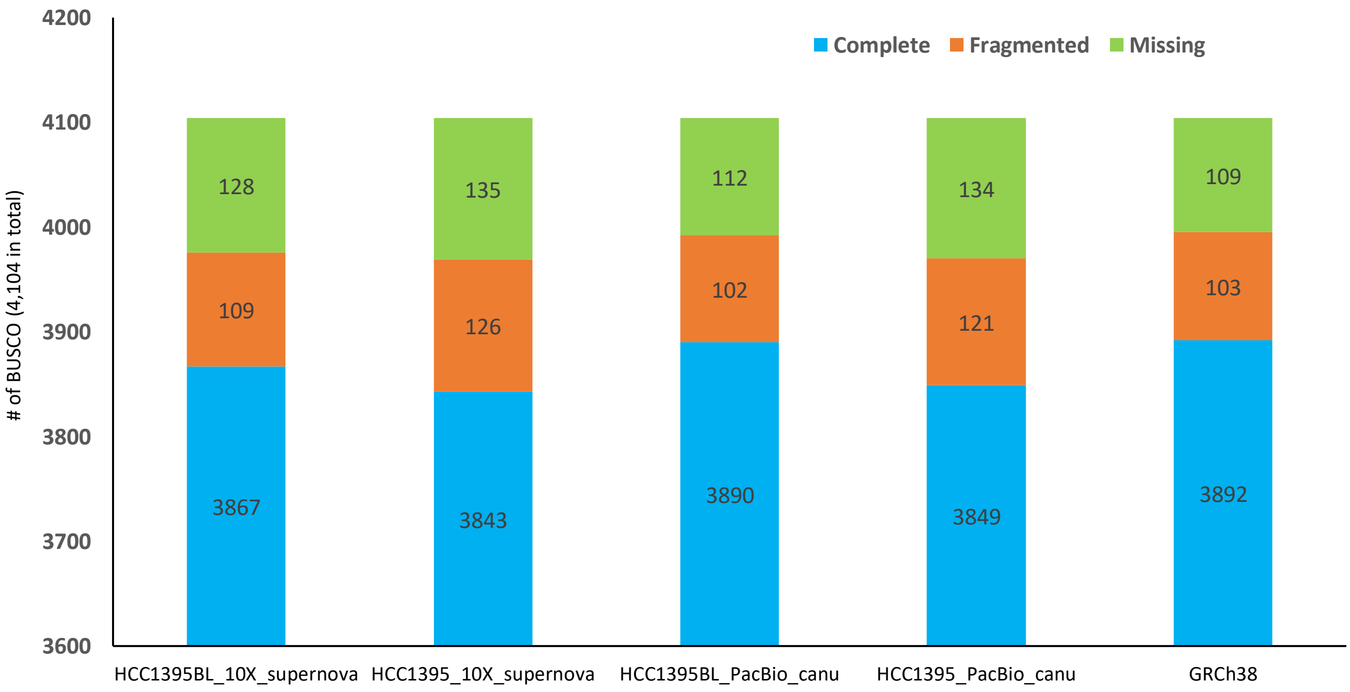

Fig. S3. BUSCO assessment of the assemblies from PacBio long reads using canu and 10X Genomics linked reads using supernova. GRCh38 (primary assembly) was listed here for comparison purposes. Lineage dataset mammalia_odb9 was used when BUSCO v3.0.0 was run in "genome" mode.

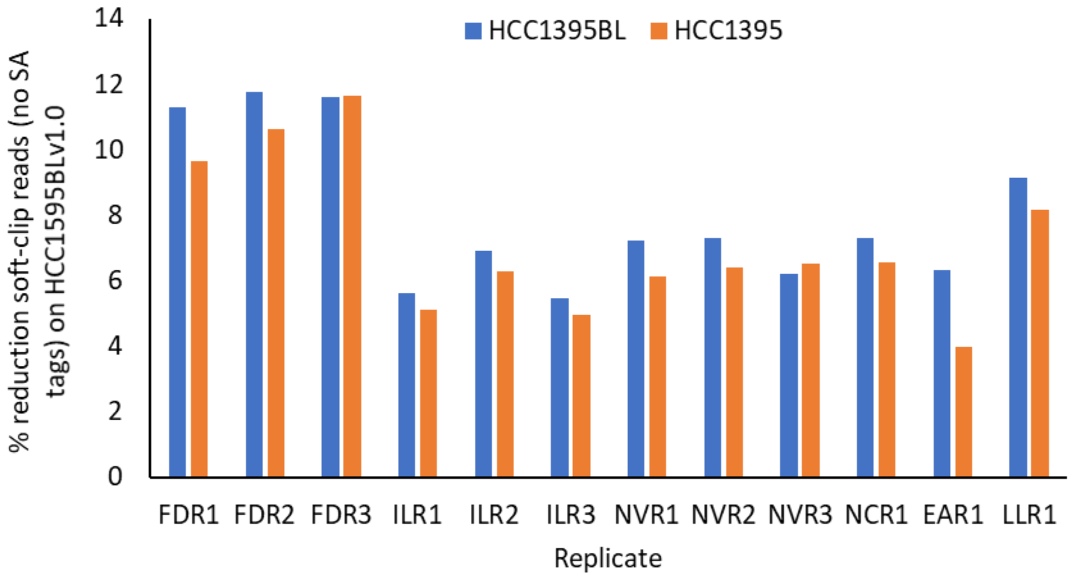

Fig. S4A. Percentages of reductions of soft-clip reads (no SA tags) with HCC1395BL_v1.0 reference and GRCh38.

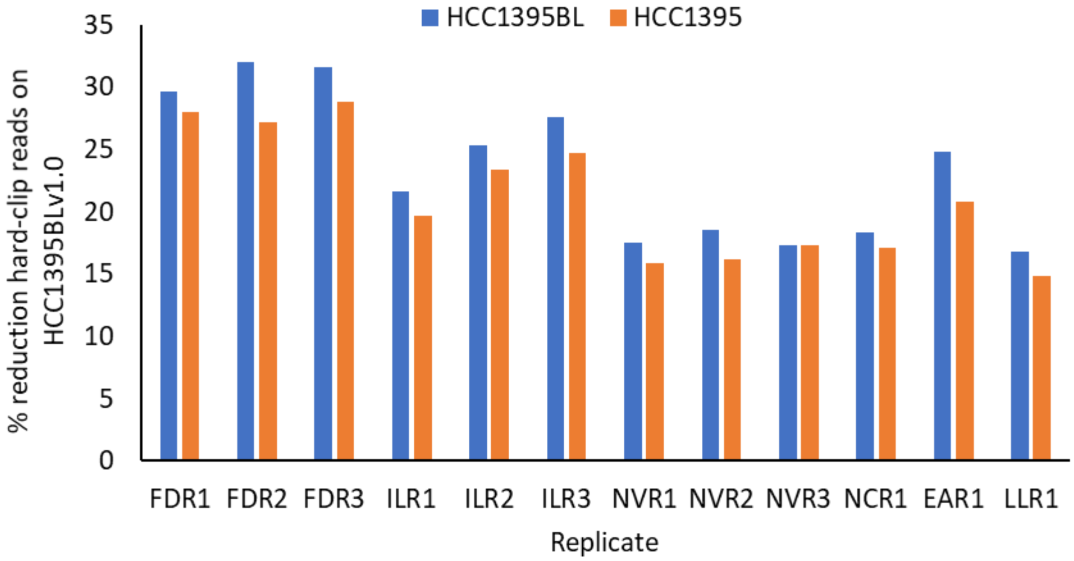

Fig. S4B. Percentages of reduction of hard-clip reads on HCC1395BL_v1.0 and GRCh38.

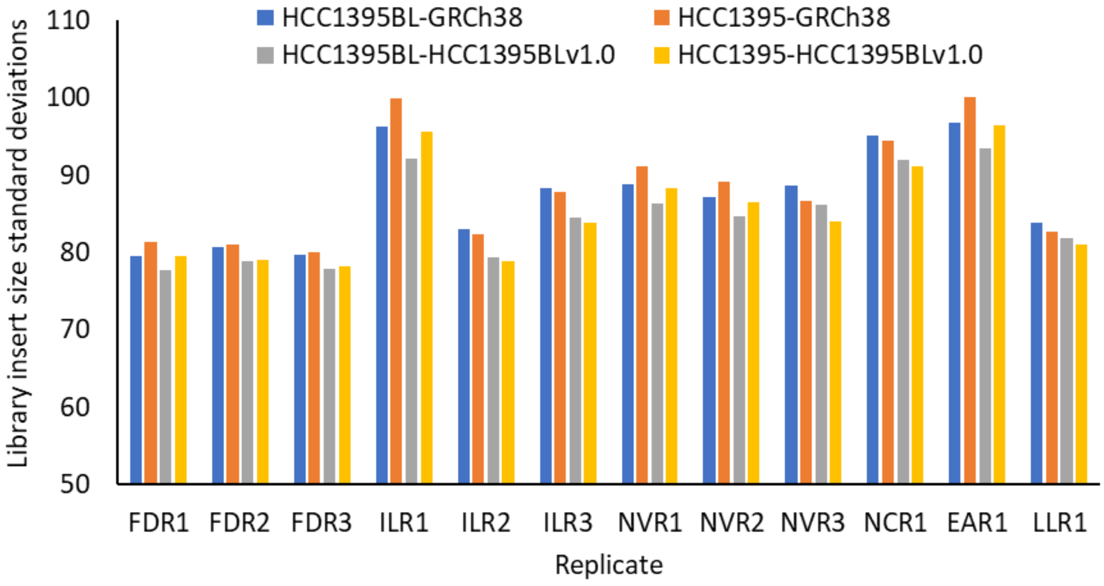

Fig. S4C. Decreases of library insert size standard deviations on HCC1395BL_v1.0 and GRCh38.

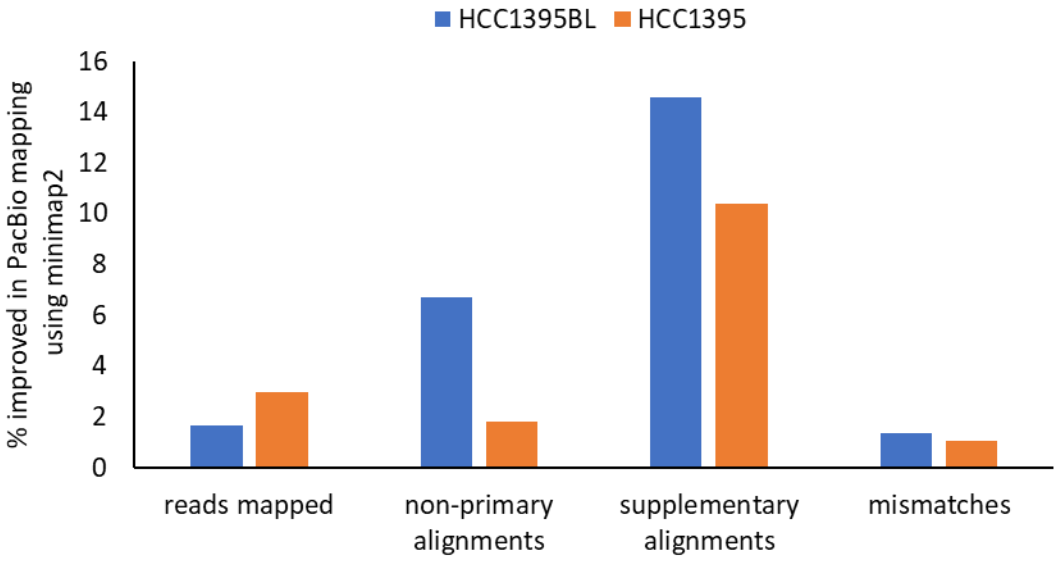

Fig. S4D. Percentages of increased read mapping rates, decreased non-primary alignments and supplementary alignments, and mismatches with HCC1395BL_v1.0 reference as opposed to GRCh38 for PacBio reads using minimap2.

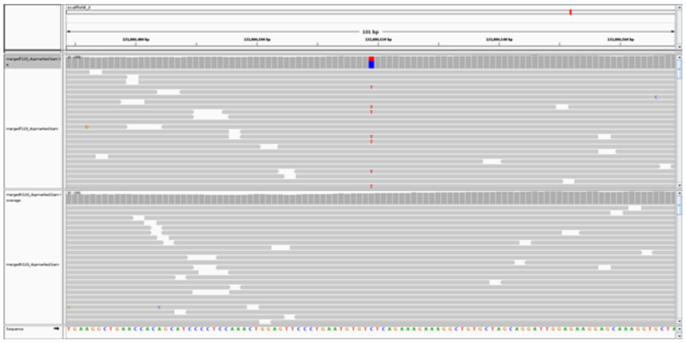

A. Intergenic SNV scaffod_2:131886519 on HCC1395BL_v1.0 assembly.

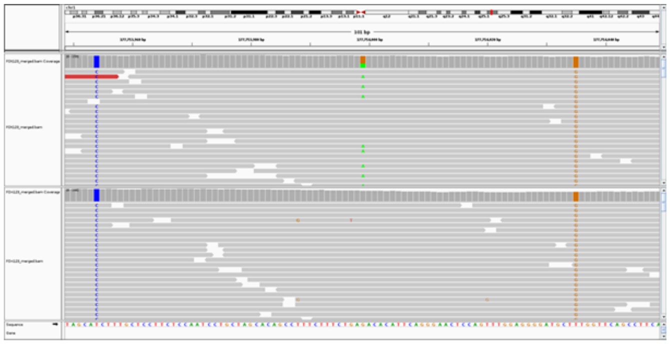

B. Intergenic SNV chr1:177753999 on GRCh38 with mismatches in flanking.

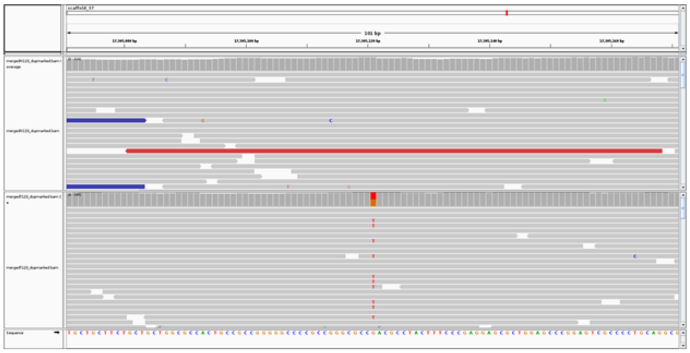

C. Exonic SNV scaffold_37: 17305121 on HCC1395BL_v1.0 assembly.

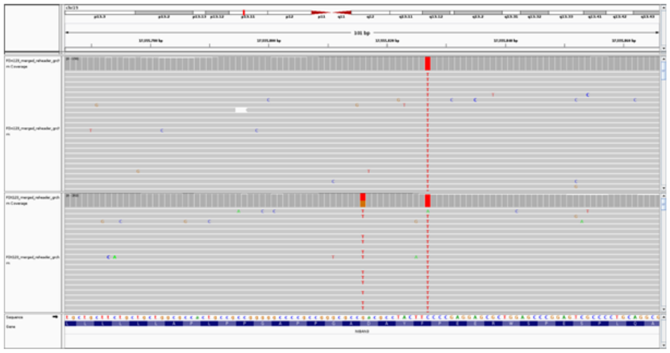

D. Exonic SNV chr19:17555816 on GRCh38 with mismatches in flanking.

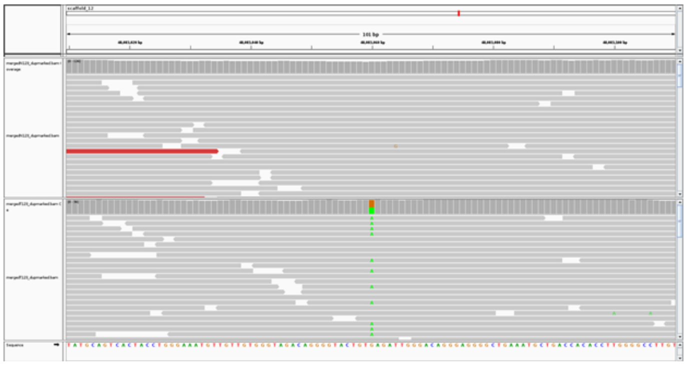

E. Intronic SNV scaffold_12:48083060 on HCC1395BL_v1.0 assembly.

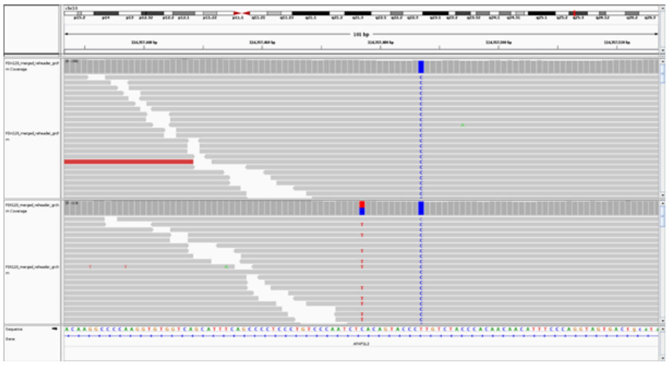

F. Intronic SNV chr10_114357477 on GRCh38 with mismatches in flanking.

```
Query= chr1_177753999_ref

Length=101
                                                                      Score     E
Sequences producing significant alignments:                          (Bits)  Value

scaffold_2                                                            176     2e-42

>scaffold_2
Length=159912780

 Score = 176 bits (95),  Expect = 2e-42
 Identities = 99/101 (98%), Gaps = 0/101 (0%)
 Strand=Plus/Minus

Query  1          TAGCATCTTTGCTCCTTCTCCAATCCTGCTAGCACAGCCTTTCTTTCTGAGACACATTCA  60
                  ||||| ||||||||||||||||||||||||||||||||||||||||||||||||||||||
Sbjct  131886569  TAGCACCTTTGCTCCTTCTCCAATCCTGCTAGCACAGCCTTTCTTTCTGAGACACATTCA  131886510

Query  61         GGGAACTCCAGTTTGGAGGGGATGCTTTGGTTCAGCCTTCA  101
                  |||||||||||||||||||||||||| ||||||||||||||
Sbjct  131886509  GGGAACTCCAGTTTGGAGGGGATGCTGTGGTTCAGCCTTCA  131886469


Query= chr1_177753999_alt

Length=101
                                                                      Score     E
Sequences producing significant alignments:                          (Bits)  Value

scaffold_2                                                            171     7e-41

>scaffold_2
Length=159912780

 Score = 171 bits (92),  Expect = 7e-41
 Identities = 98/101 (97%), Gaps = 0/101 (0%)
 Strand=Plus/Minus

Query  1          TAGCATCTTTGCTCCTTCTCCAATCCTGCTAGCACAGCCTTTCTTTCTGAAACACATTCA  60
                  ||||| |||||||||||||||||||||||||||||||||||||||||||| |||||||||
Sbjct  131886569  TAGCACCTTTGCTCCTTCTCCAATCCTGCTAGCACAGCCTTTCTTTCTGAGACACATTCA  131886510

Query  61         GGGAACTCCAGTTTGGAGGGGATGCTTTGGTTCAGCCTTCA  101
                  |||||||||||||||||||||||||| ||||||||||||||
Sbjct  131886509  GGGAACTCCAGTTTGGAGGGGATGCTGTGGTTCAGCCTTCA  131886469
```

G. BLAST alignment example of reference allele and alternate allele from SNV chr1:177753999 mapped onto de novo assembly.

Fig. S5. Illustration of GRCh38 SNV mapping to *de novo* assembly with discrepancies.
(A) IGV snapshot for an intergenic SNV scaffold_2:131886519, the alleles were reverse-complement mapped on the *de novo* assembly.
(B) IGV snapshot for SNV chr1:177753999 on GRCh38 with mismatches in flanking sequences. The same set of reads (266 reads from HCC1395BL sample, and 278 reads from HCC1395 sample) were found to align (with mapping quality 60) crossing the corresponding SNV regions (scaffold_2:131886469-131886569 for SNV scaffold_2:131886519 on HCC1395BL_v1.0; chr1:177753949-177754049 for SNV chr1:177753999 on GRCh38).
(C) IGV snapshot for an exonic SNV scaffold_37: 17305121 on HCC1395BL_v1.0 assembly.
(D) IGV snapshot for an exonic SNV chr19:17555816 on GRCh38 with mismatches in the flanking sequence. This somatic SNV causes an amino acid change (Asp -> Tyr for GAC -> TAC) in gene COLGALT1 on chr19.
(E) IGV snapshot for an intronic SNV scaffold_12:48083060 on HCC1395BL_v1.0 assembly.
(F) IGV snapshot for an intronic SNV chr10_114357477 on GRCh38 with mismatches in flanking sequence. This somatic SNV is located in the intronic region of the gene AFAP1L2 on chr10.
(G) BLAST alignment example of reference allele and alternate allele from SNV chr1:177753999 mapped onto de novo assembly with reference allele 98% identity and 101 bps alignment for reference allele, and 97% identity and 101 bps alignment for alternate allele.

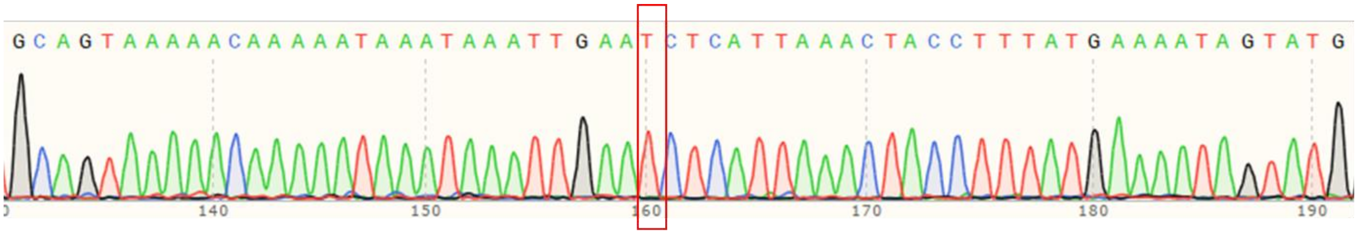

Normal sample HCC1395BL with a homozygous site (T/T) on scaffold_17:32958026

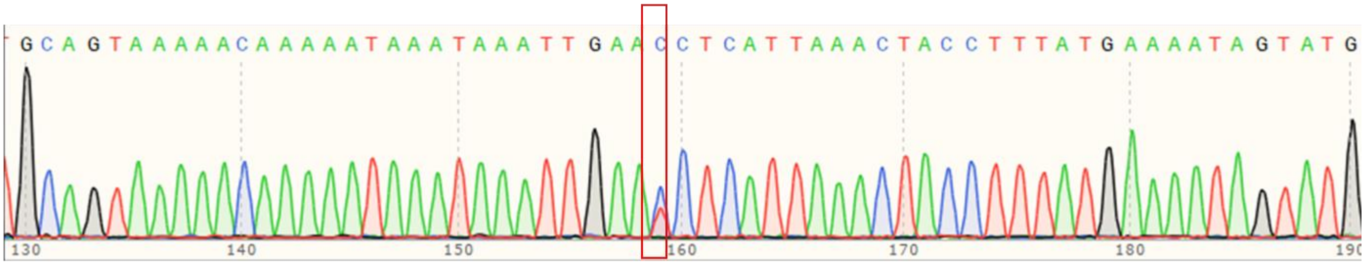

Tumor sample HCC1395 with a heterozygous site (T/C) on scaffold_17:32958026

**PCR primers and sequences**	**scaffold_17_32958026_F1: GGGATCGC TATCTCTCACCA**<br>**scaffold_17_32958026_F2: TGCATTTG TGAATTGTGCTG**
**Somatic SNV scaffold_17_32958026 (MAF=0.705) and its flanking**	**AAATGCAAATGCAGTAAAAACAAAAATAAATAAATTGAA [T/C] CTCATTAAACTACCTTTATGAAAATAGTATGGAGCTTTCTCAAG**

Fig. S6. An example of PCR validation using Sanger sequencing for a SNV site, scaffold_17:32958026, at which a somatic mutation from T to C was confirmed.

A

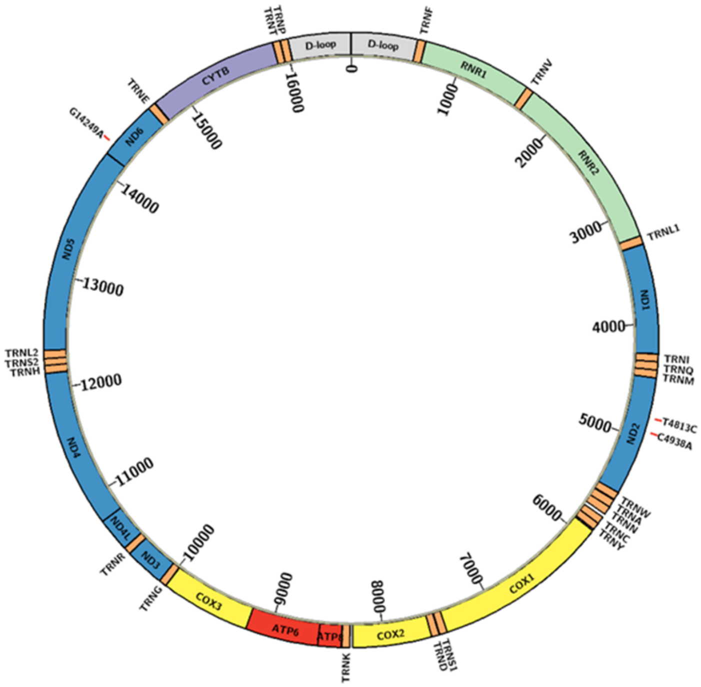

B

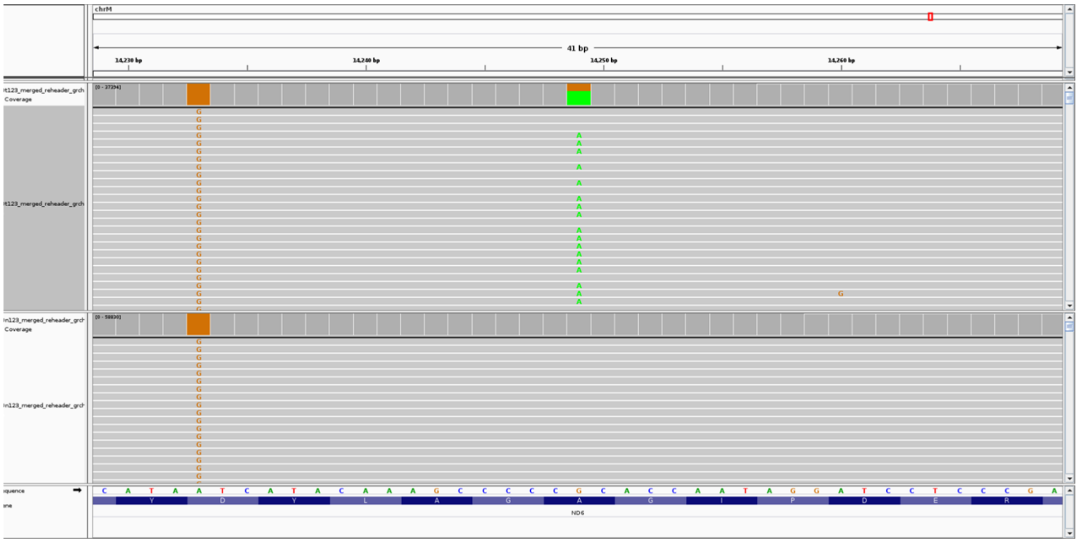

Fig. S7. (A) Mitochondrial genome of HCC1395BL/HCC1395 with three unreported non-synonymous somatic mutations (T4813C and C4938A on MT-ND2 gene, and G14249A on MT-ND6 gene), which were verified on IGV for G14249A (as an example) in the HCC1395 cell line only, but not in the HCC1395BL cell line (B).

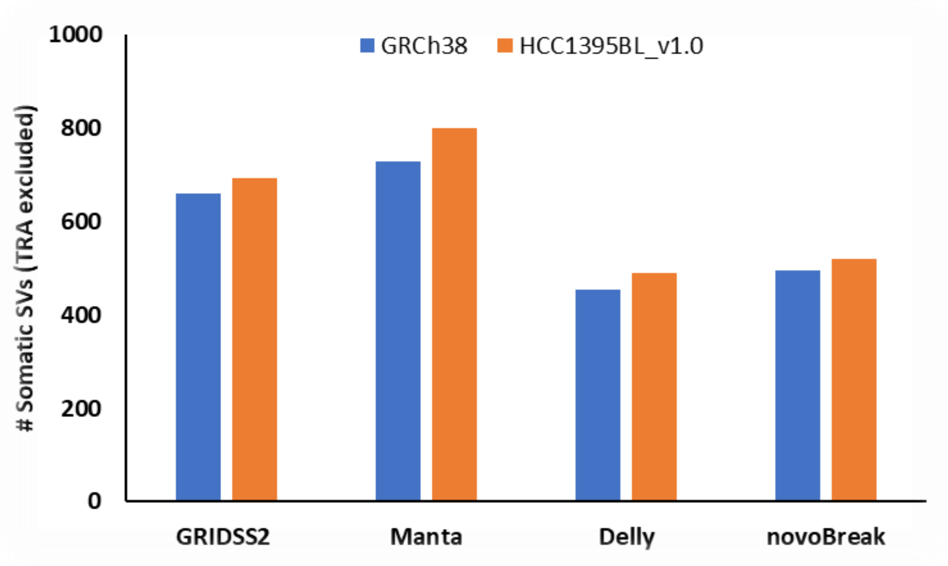

Fig. S8A. Somatic SVs (TRA excluded) detected by 4 somatic SV callers with HCC1395BL_v1.0 reference as compared to GRCh38 with short-read sequencing data from a paired sample (FDT123/FDN123).

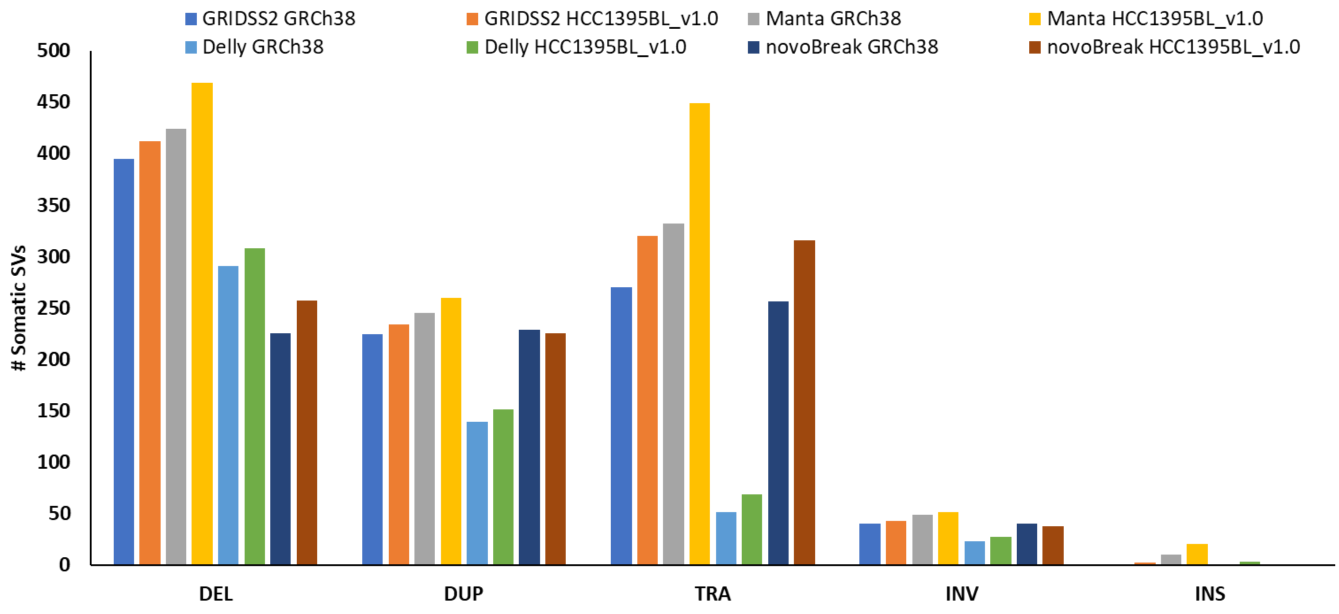

Fig. S8B. Somatic SVs by SV types detected by 4 somatic SV callers with HCC1395BL_v1.0 reference as compared to GRCh38 with short-read sequencing data from a paired sample (FDT123/FDN123).

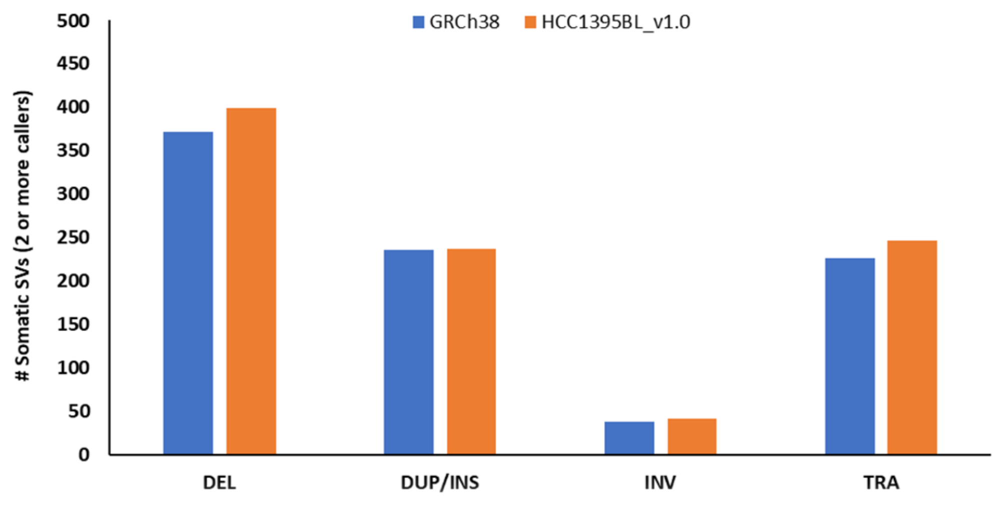

Fig. S8C. The counts of somatic SVs that were supported by 2 or more SV callers with HCC1395BL_v1.0 reference as compared to GRCh38 using short-read sequencing data from a paired sample (FDT123/FDN123).

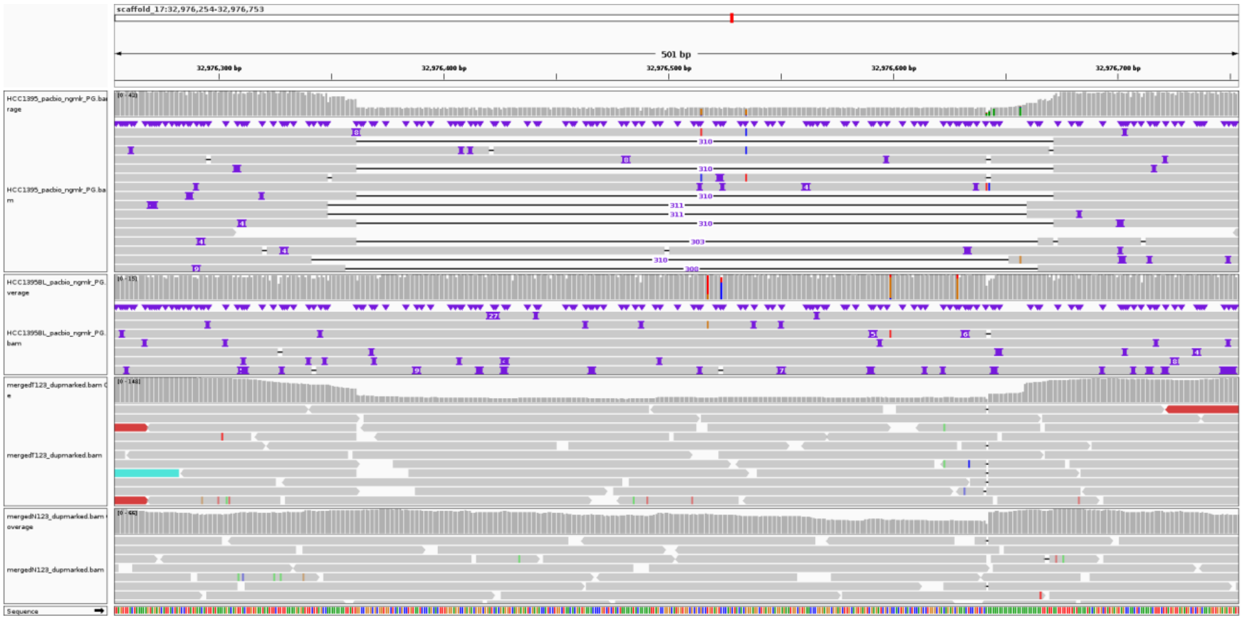
scaffold_17:32,976,254-32,976,753
501 bp
32,976,300 bp
32,976,400 bp
32,976,500 bp
32,976,600 bp
32,976,700 bp
Sequence

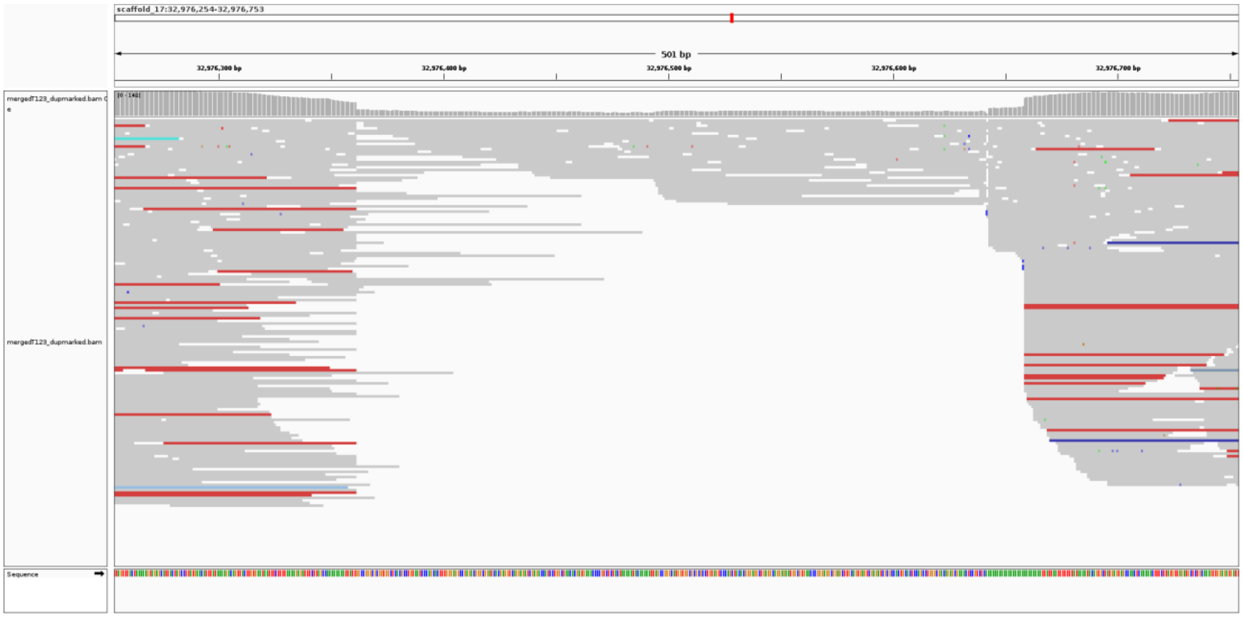
scaffold_17:32,976,254-32,976,753
501 bp
32,976,300 bp
32,976,400 bp
32,976,500 bp
32,976,600 bp
32,976,700 bp
mergedT123_dupmarked.bam
Sequence

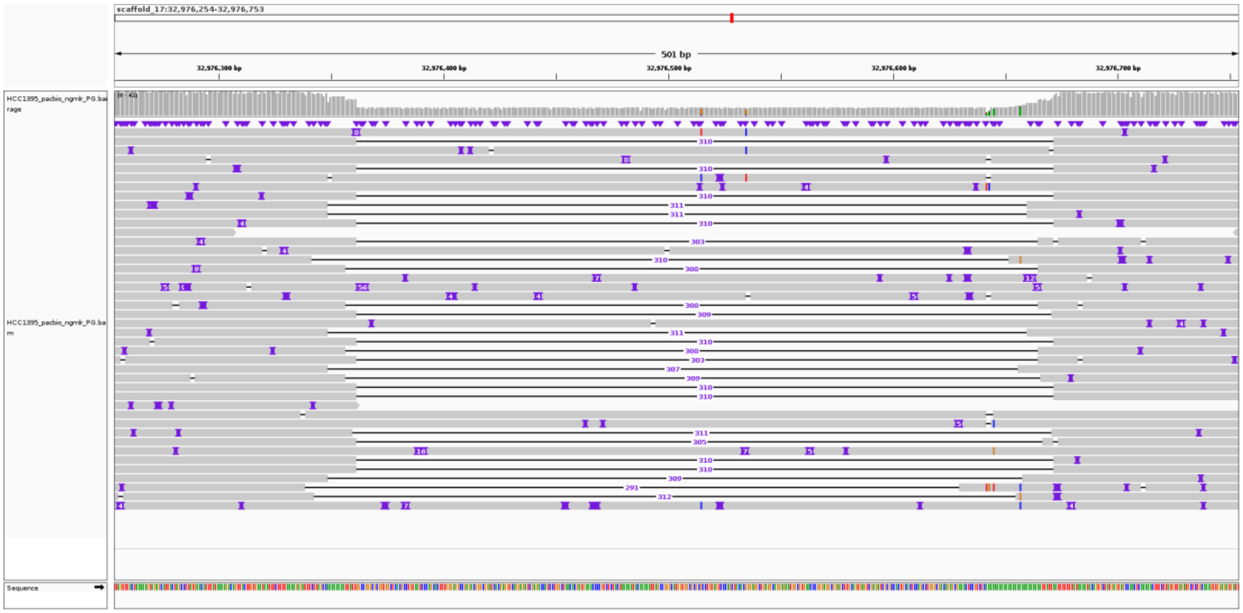

Fig. S9A. A 311 base pair *SINE/Alu* heterozygous deletion (scaffold_17:32976348-32976659) in cancer cell line, which overlaps with CCDC91 (coiled-coil domain containing 91) gene, was discovered with Illumina short-reads when using HCC1395BL_v1.0 reference, but not when using GRCh38 with the same set of Illumina short reads due to the absence of this *Alu* sequence (Alignment tracks in IGV: NGMLR-tumor-pacbio, NGMLR-normal-pacbio, bwamem-tumor-ILMN, bwamem-normal-ILMN, respectively) (IGV snapshot at top). Full set of Illumina short reads (IGV snapshot at middle) and PacBio long reads (IGV snapshot at bottom) were shown for verification of this deletion.

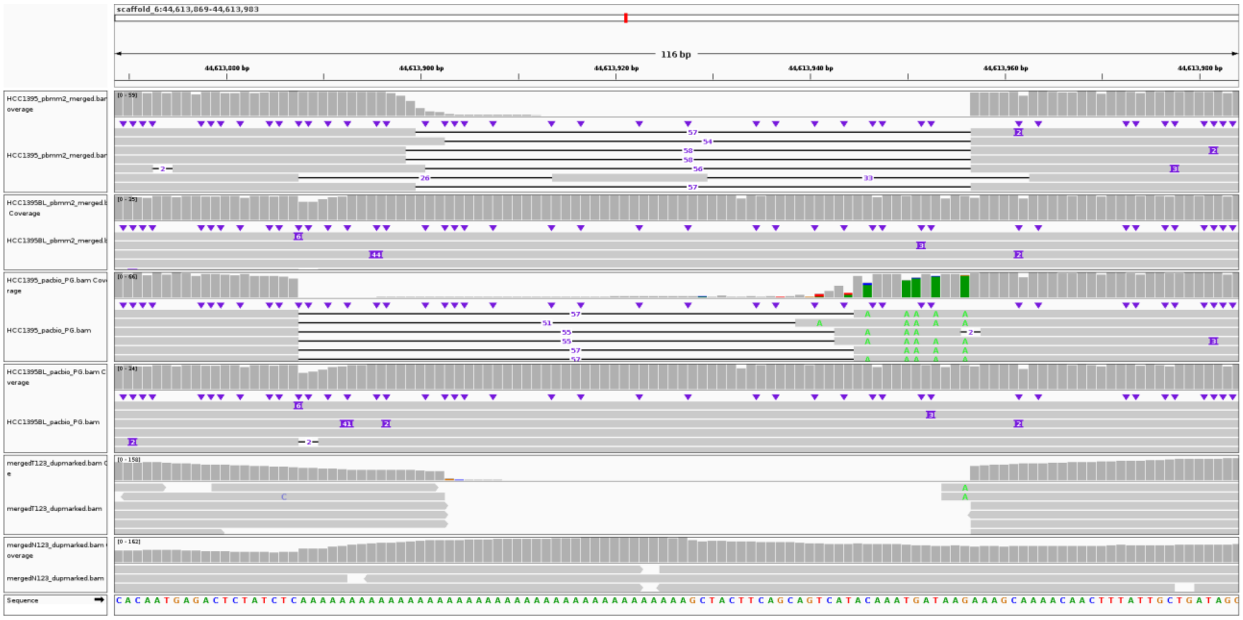

Fig. S9B. A 57 base pair homozygous deletion (scaffold_6:44613899-44613956) in cancer cell line, which overlaps with MED12L (mediator complex subunit 12L) gene, was detected with Illumina short-reads with HCC1395BL_v1.0 reference, but only a 40 base pair deletion was discovered when using GRCh38 with the same set of Illumina short reads due to 17 more adenine (A) nucleotides in this deletion region on HCC1395BL_v1.0 reference (Alignment tracks in IGV: PBMM2-tumor, PBMM2-normal, NGMLR-tumor, NGMLR-normal, bwamem-tumor, bwamem-normal, respectively). PacBio long reads were presented here for verification of this in-read deletion.

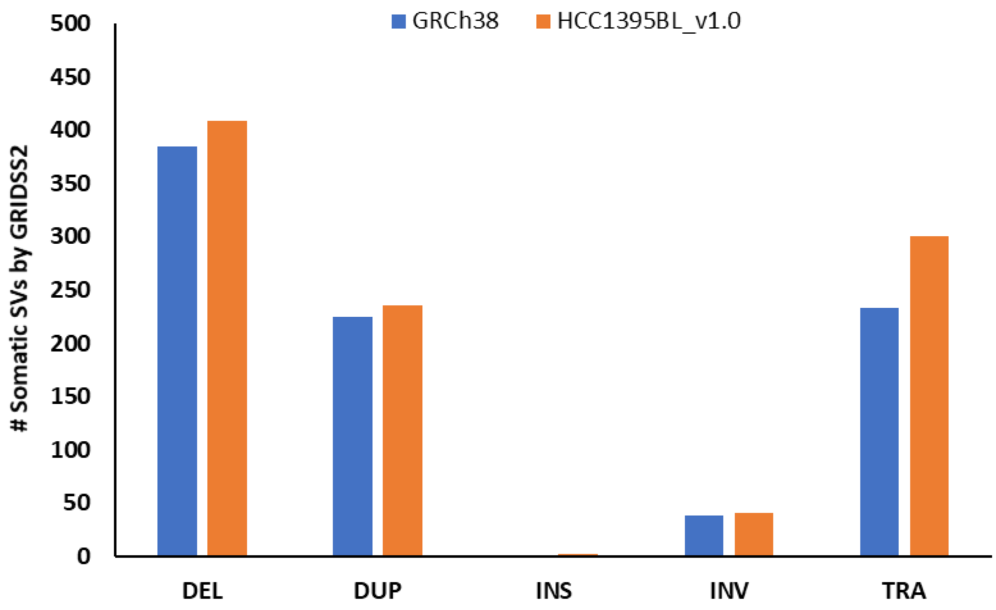

Fig. S10A. Summary of somatic SVs by SV types detected in two or more replicates by GRIDSS2 with HCC1395BL_v1.0 reference as compared to GRCh38.

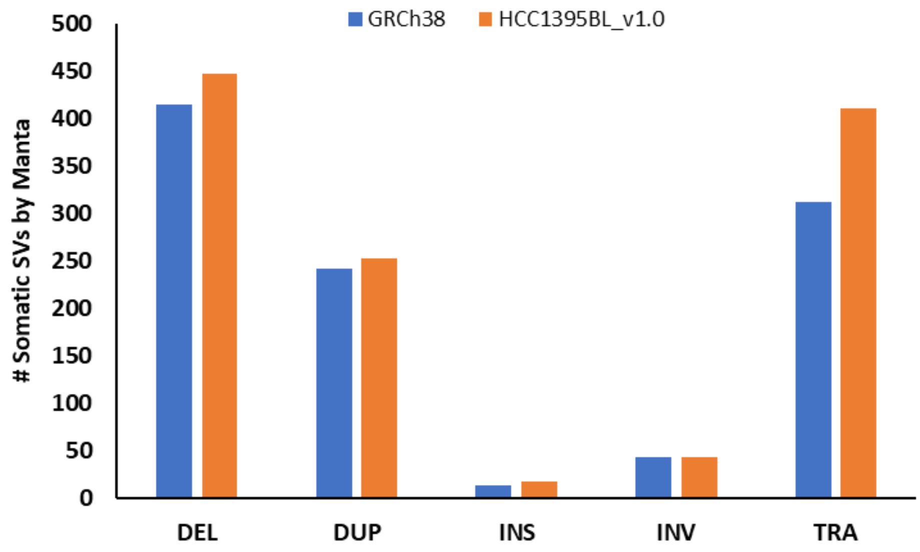

Fig. S10B. Summary of somatic SVs by SV types detected in two or more replicates by Manta with HCC1395BL_v1.0 reference as compared to GRCh38.

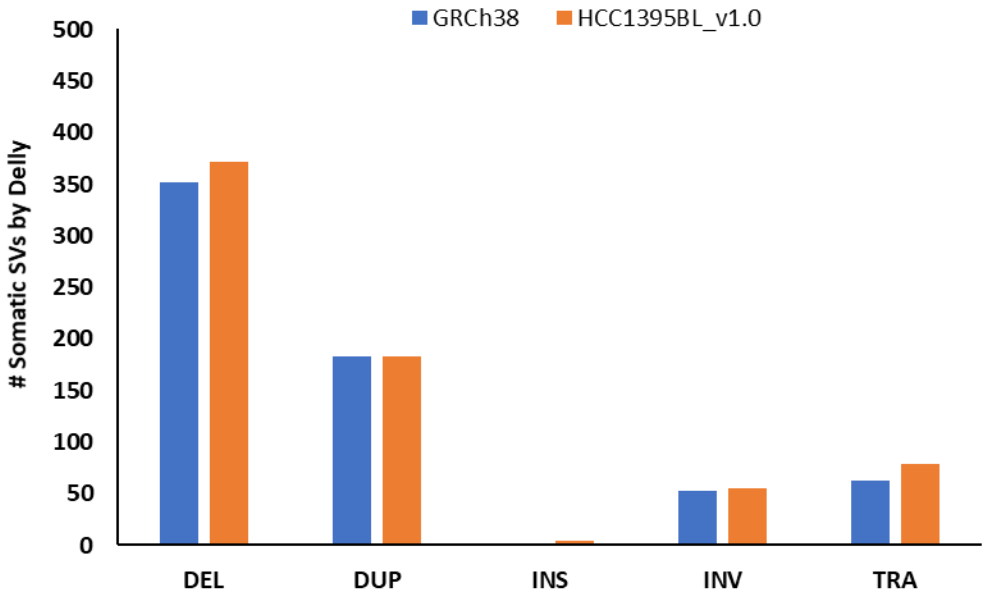

Fig. S10C. Summary of somatic SVs by SV types detected in two or more replicates by Delly with HCC1395BL_v1.0 reference as compared to GRCh38.

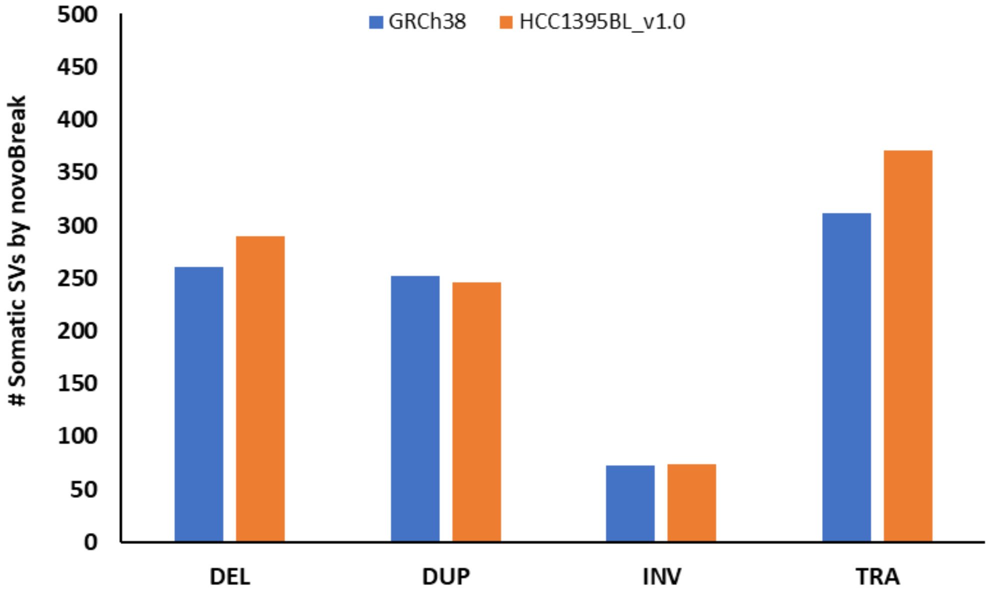

Fig. S10D. Summary of somatic SVs by SV types detected in two or more replicates by novoBreak with HCC1395BL_v1.0 reference as compared to GRCh38.

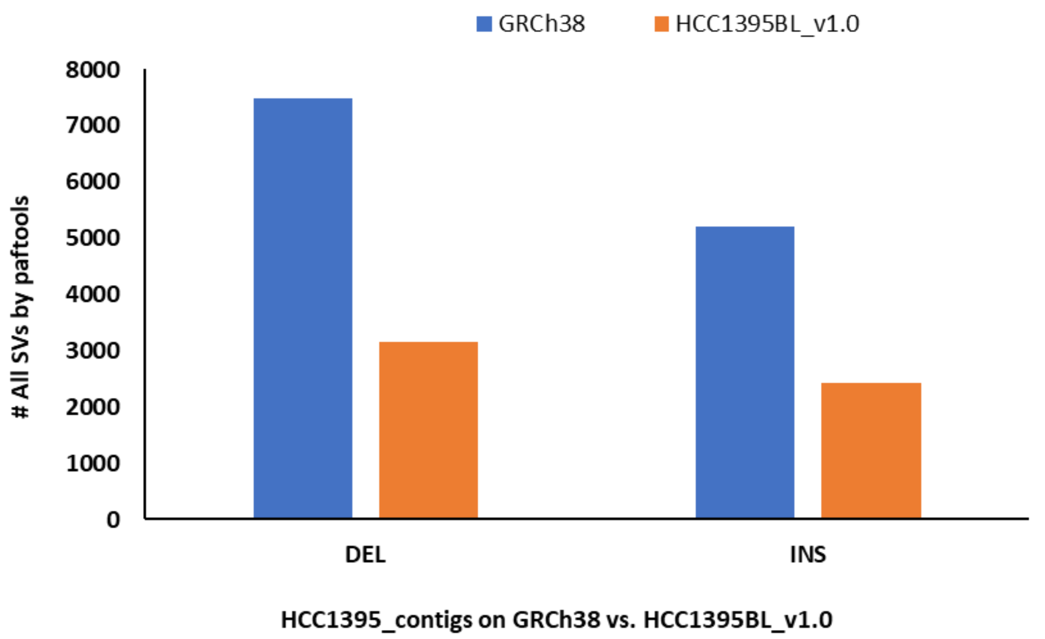

Fig. S11A. Summary of all raw SVs initially identified by paftools based on HCC1395 contigs mapped to HCC1395BL_v1.0 and GRCh38 references.

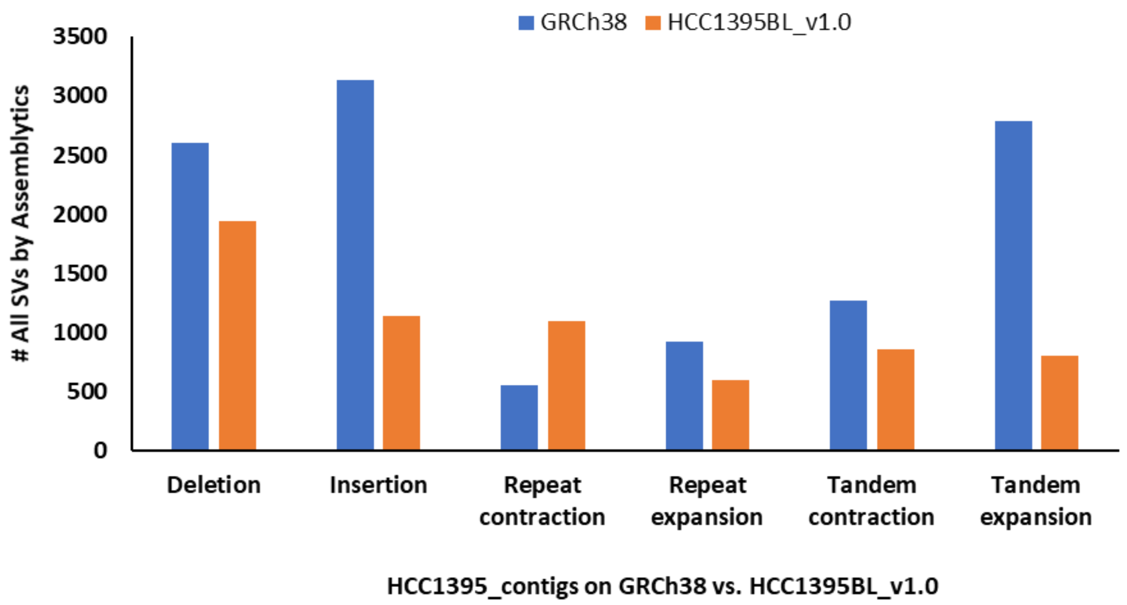

Fig. S11B. Summary of all raw SVs initially identified by Assemblytics based on HCC1395 contigs mapped to HCC1395BL_v1.0 and GRCh38 references.

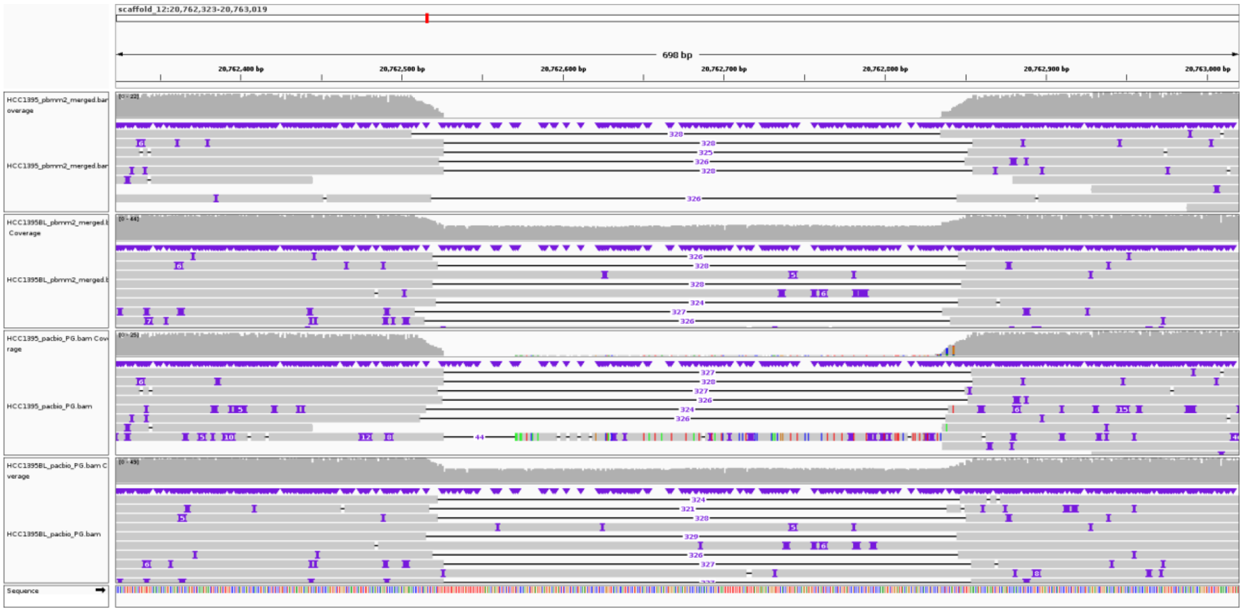

Fig. S12A. A 327 base pair homozygous deletion (scaffold_12:20762508-20762835) in cancer cell line, which was overlapping with CDH23 gene, was discovered with PacBio long reads when using HCC1395BL_v1.0 reference, but not when using GRCh38 with the same set of PacBio long reads due to the absence of the *AluY* sequence in this location (Alignment tracks in IGV: PBMM2-tumor, PBMM2-normal, NGMLR-tumor, NGMLR-normal, respectively).

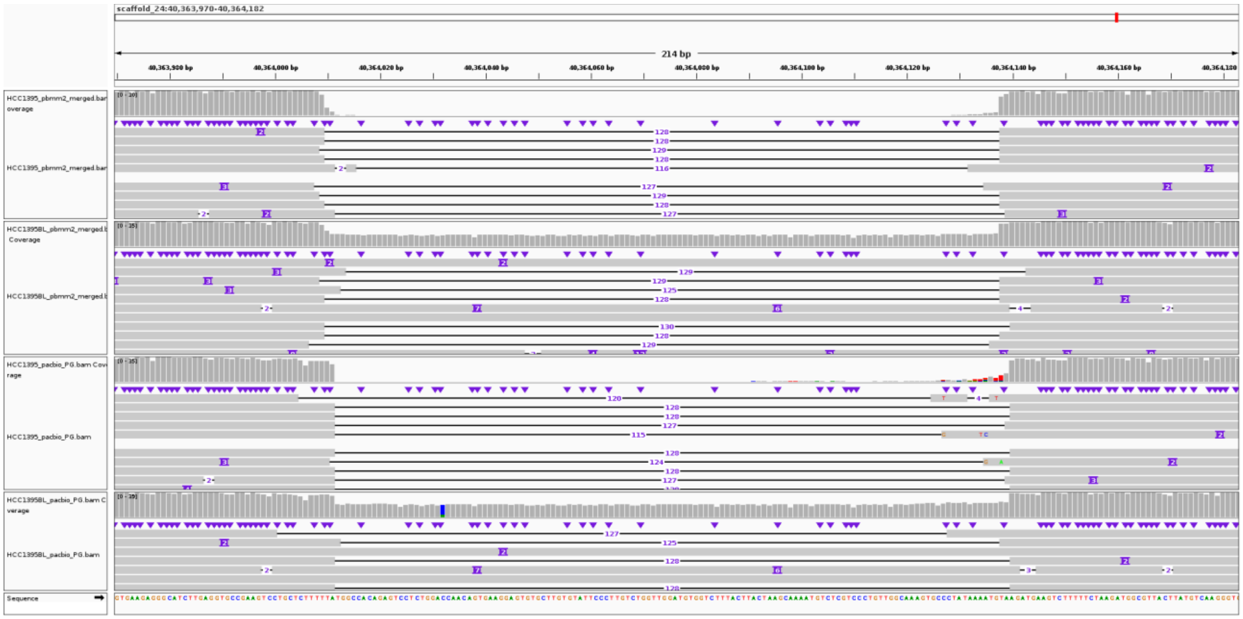

Fig. S12B. A 128 base pair homozygous deletion (scaffold_24:40364012-40364140) that overlapped an *LTR/ERVL* repeat was uncovered in tumor cell line with PacBio long reads when using HCC1395BL_v1.0 reference, but not when using GRCh38 with the same set of PacBio long reads. This deletion was located in an intronic region (exon1 and exon2) of ST14 (ST14 transmembrane serine protease matriptase). Studies have associated the expression of this protease with breast, colon, prostate, and ovarian tumors (Alignment tracks in IGV: PBMM2-tumor, PBMM2-normal, NGMLR-tumor, NGMLR-normal, respectively).

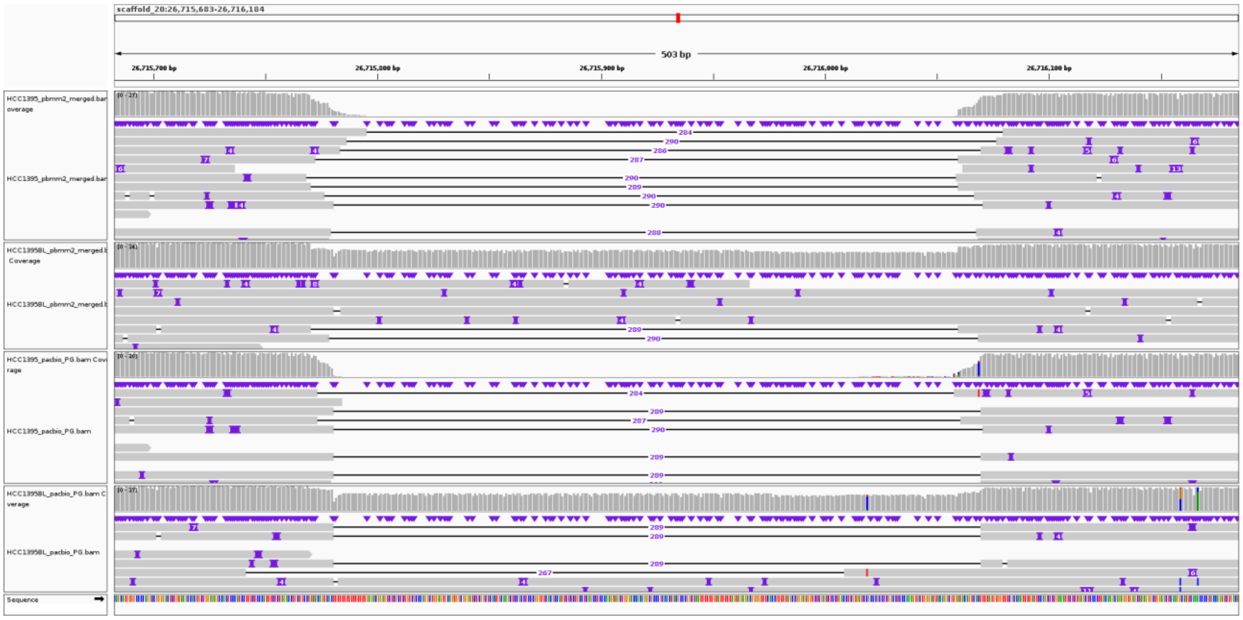

Fig. S12C. an intronic 289 base pairs homozygous *AluY* deletion (scaffold_20:26715781-26716070, between exon 16 and exon 17) in ACE (Angiotensin I converting enzyme) was identified with PacBio long reads in tumor cell line using HCC1395BL_v1.0 reference. This deletion was not detected using the GRCh38 reference with the same set of PacBio long reads, as it lacks this *Alu* sequence. Previous studies demonstrated that the presence or absence of a 287 bp *Alu* repeat element in this gene associated with circulating levels of this enzyme (Alignment tracks in IGV: PBMM2-tumor, PBMM2-normal, NGMLR-tumor, NGMLR-normal, respectively).

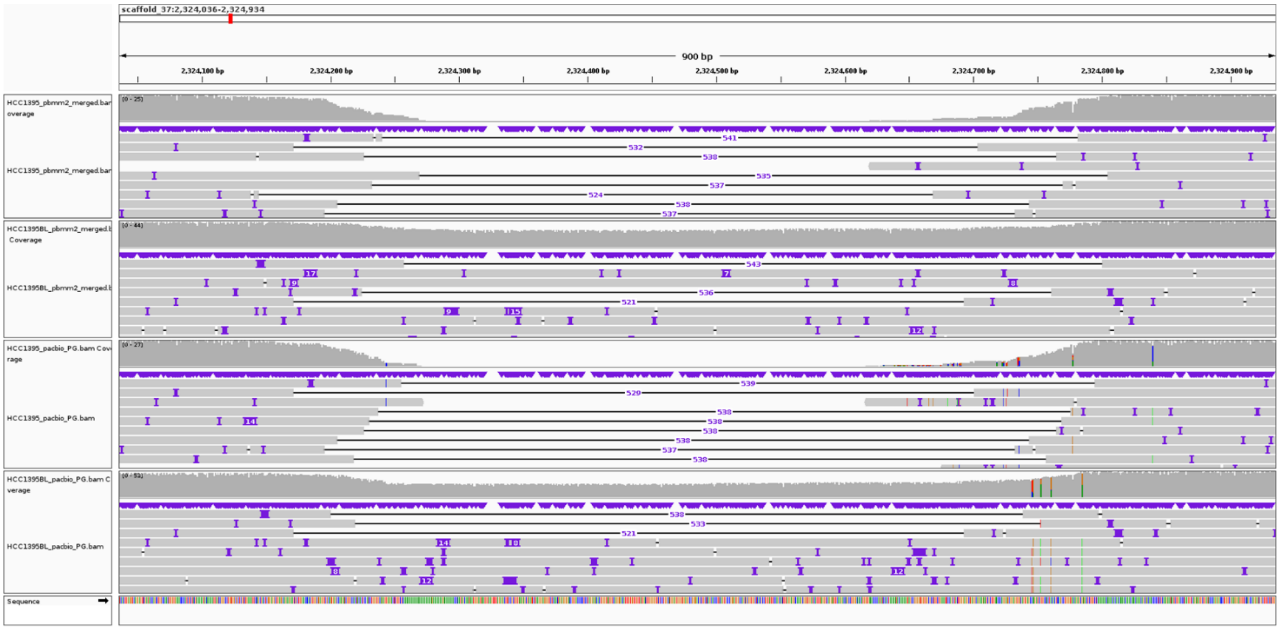

Fig. S12D. A 538 base pairs homozygous deletion (scaffold_37:2324216-2324754) was detected with PacBio long reads in the tumor cell line when using the HCC1395BL_v1.0 reference, but not when using GRCh38 with the same set of PacBio long reads. The sequence of this deletion overlaps a *SINE/Alu* repeat (*AluSx*) in an intronic region of GNG7 (G protein subunit gamma 7), which may be associated with G protein-coupled receptor signaling pathway and regulation of adenylate cyclase activity (Alignment tracks in IGV: PBMM2-tumor, PBMM2-normal, NGMLR-tumor, NGMLR-normal, respectively).

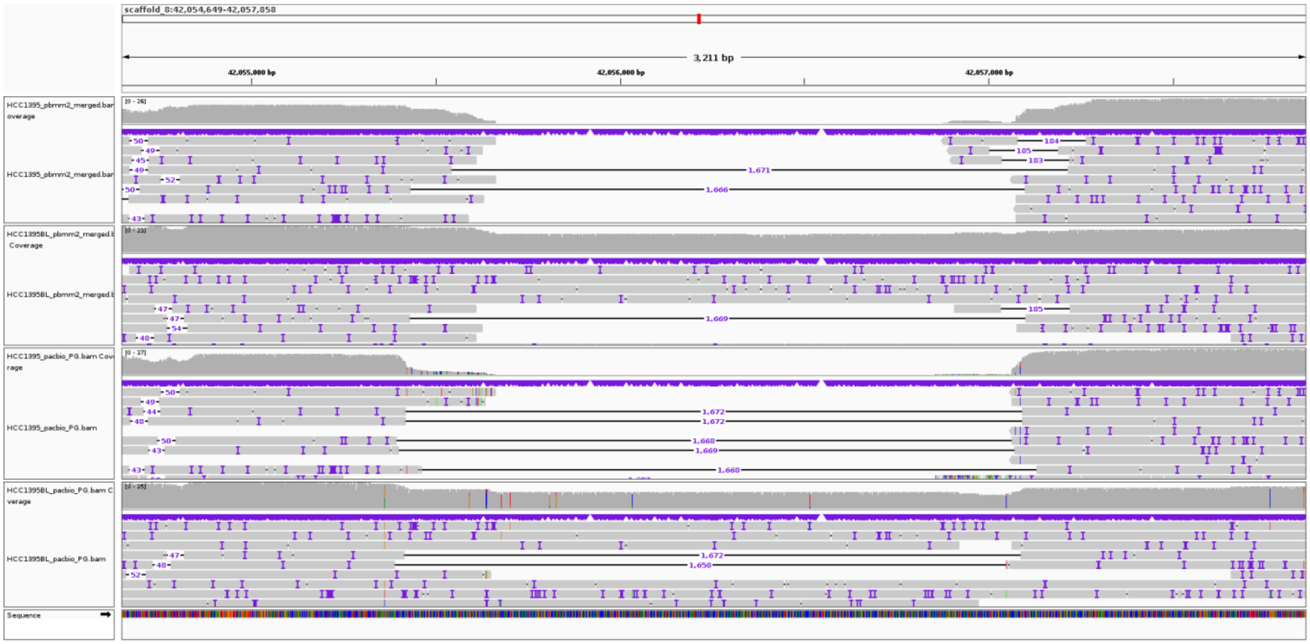

Fig. S12E. A 1,672 base pair homozygous deletion (scaffold_8:42055418-42057090) was detected with PacBio long reads in the tumor cell line when using the HCC1395BL_v1.0 reference. This deletion was located in the region of JAG2 (Jagged canonical Notch ligand 2) gene, which encodes transmembrane receptors that are critical for various cell fate decisions. This deletion was not detected using GRCh38 reference with the same set of PacBio long reads, and the sequences of this deletion were not annotated with repeats by RepeatMasker (Alignment tracks in IGV: PBMM2-tumor, PBMM2-normal, NGMLR-tumor, NGMLR-normal, respectively).

A

	# somatic SVs on GRCh38 with 2 or more calling methods	# somatic SVs on HCC1395BL_v1.0 with 2 or more calling methods	# somatic SVs on GRCh38 with 3 or more calling methods	# somatic SVs on HCC1395BL_v1.0 with 3 or more calling methods
**DEL**	939	1,102	655	763
**DUP/INS**	593	623	305	336
**INV**	63	71	35	38
**TRA**	274	322	142	137
**Total**	**1,869**	**2,118**	**1,137**	**1,274**

B

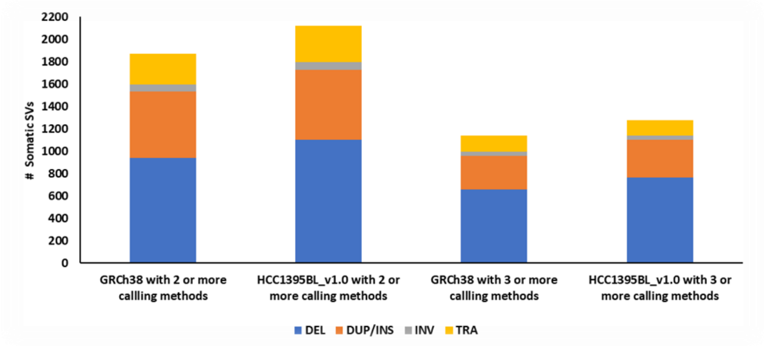

C

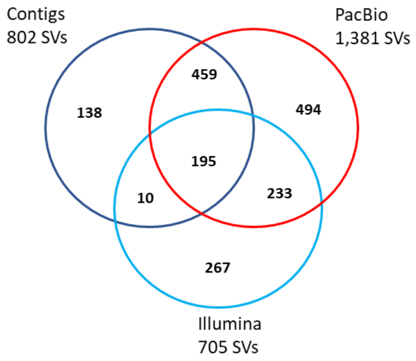

Fig. S13. Summary of consensus somatic SV calls based on 4 short-read callsets, 4 long-read callsets, and 2 contig callsets with HCC1395BL_v1.0 reference as compared to GRCh38. (A) Numbers of merged somatic SVs by SV types in a tabular format; (B) Numbers of merged somatic SVs by SV types in bar graph; (c) Venn diagram showing the consistency between somatic SV sets that were generated from three major data sources (including Illumina short-reads, PacBio long-reads, and assembled contigs for the tumor cell line), requiring each of the somatic SVs to be supported by two or more calling methods (TRA excluded).
